# Supplementary material for: Substrate stiffness dictates unique paths towards proliferative arrest in WI-38 cells
Source: GeroScience. 2025 Sep 20;48(3):4173–93. doi: 10.1007/s11357-025-01858-5 (PMC13356154; doi:10.1007/s11357-025-01858-5)
Supplement: Supplementary file 5 — Supplementary file5 (PDF 43449 KB) [file 11357_2025_1858_MOESM5_ESM.pdf]

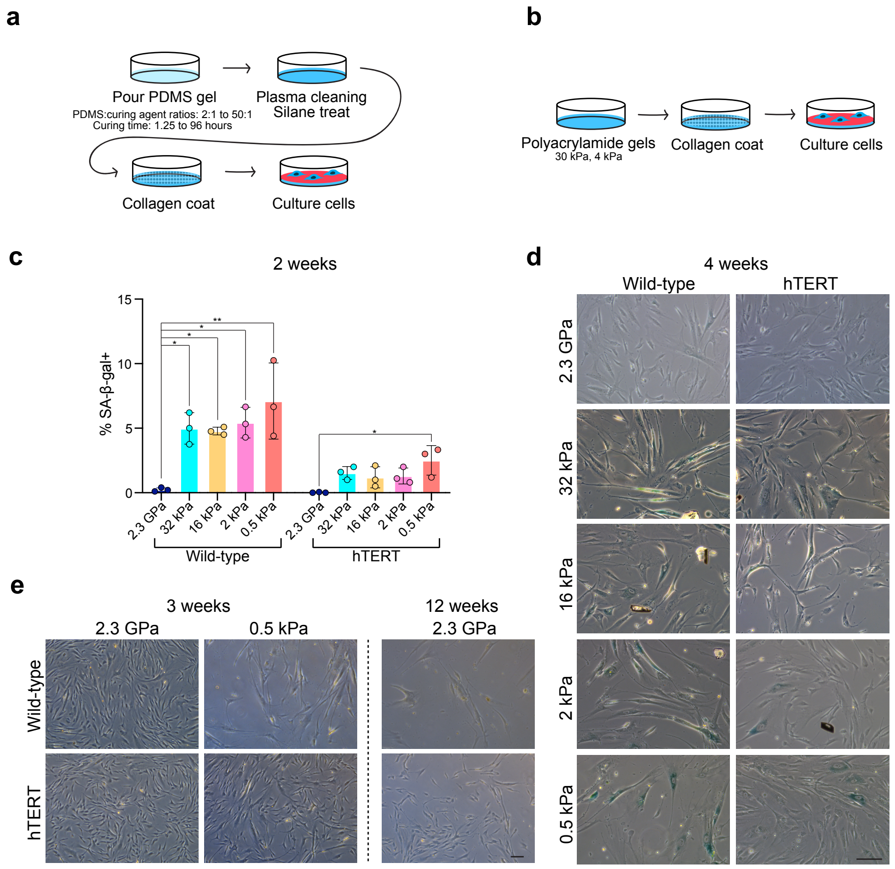

Figure S1: Wild-type WI-38 cells on soft matrices exhibit classic features of senescent cells.

(a) Schematic depicting PDMS plate fabrication. (b) Schematic for polyacrylamide gel experiments. (c) Bar graphs quantifying the percent of cells that stained for SA-β-gal in wild-type and hTERT-expressing WI-38 cells after 2 weeks in culture. (d) Representative images of SA-β-gal staining after 4 weeks of culture. (e) Representative images of wild-type and hTERT cells on the 2.3 GPa and 0.5 kPa surfaces at the indicated time points.

Bar graphs are mean ± standard deviation. 3 replicates were performed per condition (c). P values were calculated by ordinary one-way ANOVA with Tukey's multiple comparisons test (c). $^*P < 0.05$, $^{**}P < 0.01$. Scale bars, 100 μm.

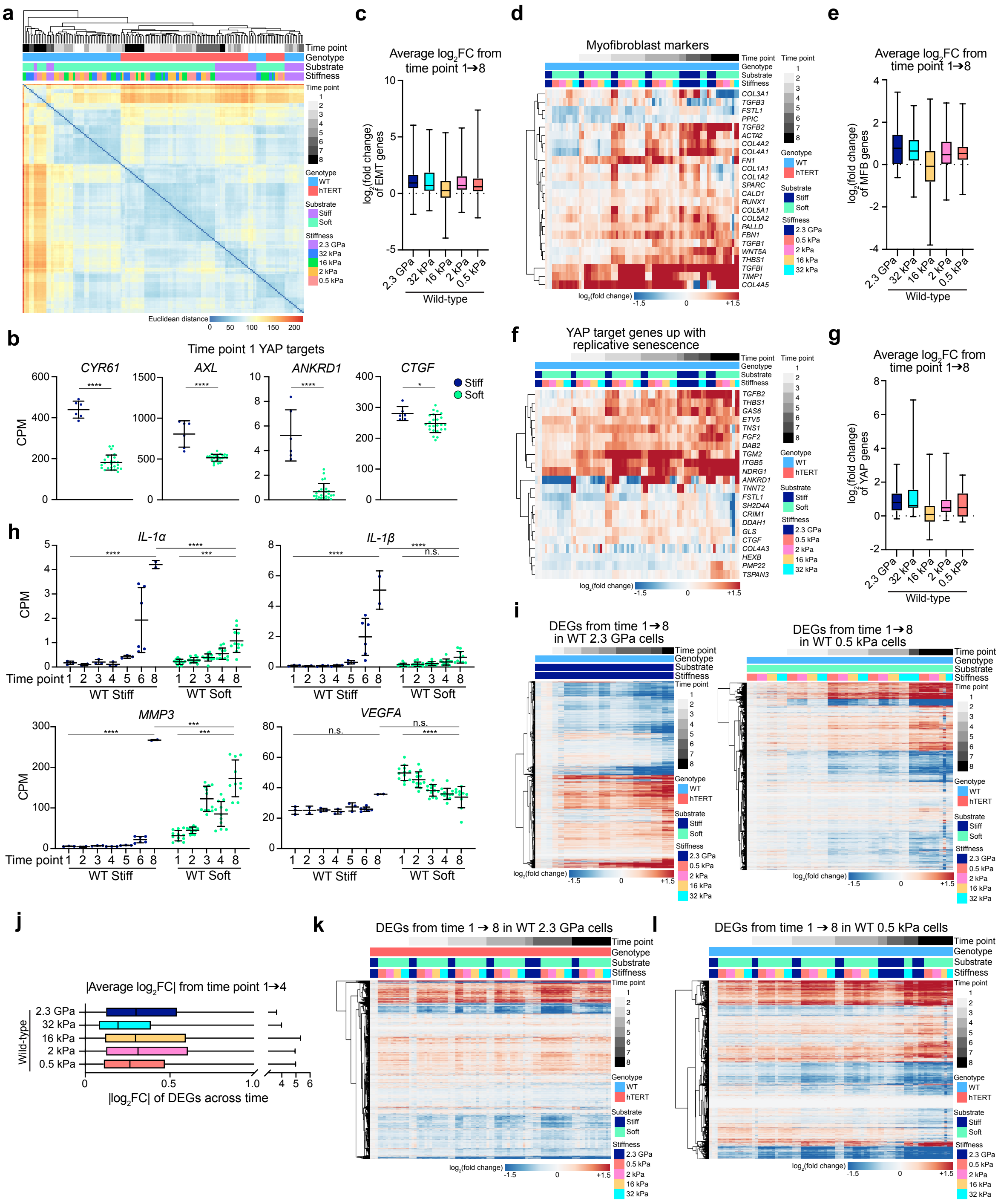

Figure S2: WI-38 cell gene expression programs are similar across substrate stiffnesses. (a) Heatmap of the Euclidean distance between RNA-sequencing samples. (b) Dot plots comparing the expression of YAP target genes in WI-38 cells on stiff and soft matrices at time point 1. (c) Box plots quantifying the $\log_2$(fold change) of each gene in (Fig. 2d) from time point 1 to time point 8 averaged across replicates. (d) Heatmap of the $\log_2$(fold change) of myofibroblast (MFB) markers previously identified[7] as upregulated in replicative senescence. (e) Box plots

quantifying the log_2(fold change) of each gene in (d) from time point 1 to time point 8 averaged across replicates. (f) Heatmap of the log_2(fold change) of YAP target genes previously identified[7] as upregulated in replicative senescence. (g) Box plots quantifying the log_2(fold change) of each gene in (f) from time point 1 to time point 8 averaged across replicates. (h) Dot plots comparing the expression of SASP genes[22] in wild-type WI-38 cells on the indicated surfaces across time. (i) Heatmap from (Fig. 2f) split by wild-type cells grown on (left) stiff or (right) soft substrates. (j) Box plots quantifying the absolute value of the log_2(fold change) of each gene in (Fig. 2f) from time point 1 to time point 4 averaged across replicates. (k) Heatmap of the log_2(fold change) of genes from (Fig. 2f) in hTERT samples across time. (l) Heatmap of the log_2(fold change) of all DEGs in wild-type cells grown on the 0.5 kPa matrix from time point 1 to time point 8 plotted across all wild-type samples across time.

Dot plots show mean ± standard deviation. Box plots show median ± 25th and 75th percentiles, with whiskers showing minimum to maximum. 6 replicates were used for the stiff substrate and 24 replicates were used for the soft substrate in (b). 3 replicates were used for the stiff time point 1, 3, and 4 conditions, 2 replicates were used for the stiff time point 2 and 8 conditions, 12 replicates were used for the soft time point 1, 2, 3, and 4 conditions, and 10 replicates were used for the soft time point 8 condition (h). P values were calculated by two-tailed Student's t-test (b) or ordinary one-way ANOVA with Tukey's multiple comparisons test (h). *$P < 0.05$, ***$P < 0.001$, ****$P < 0.0001$. n.s., not significant.

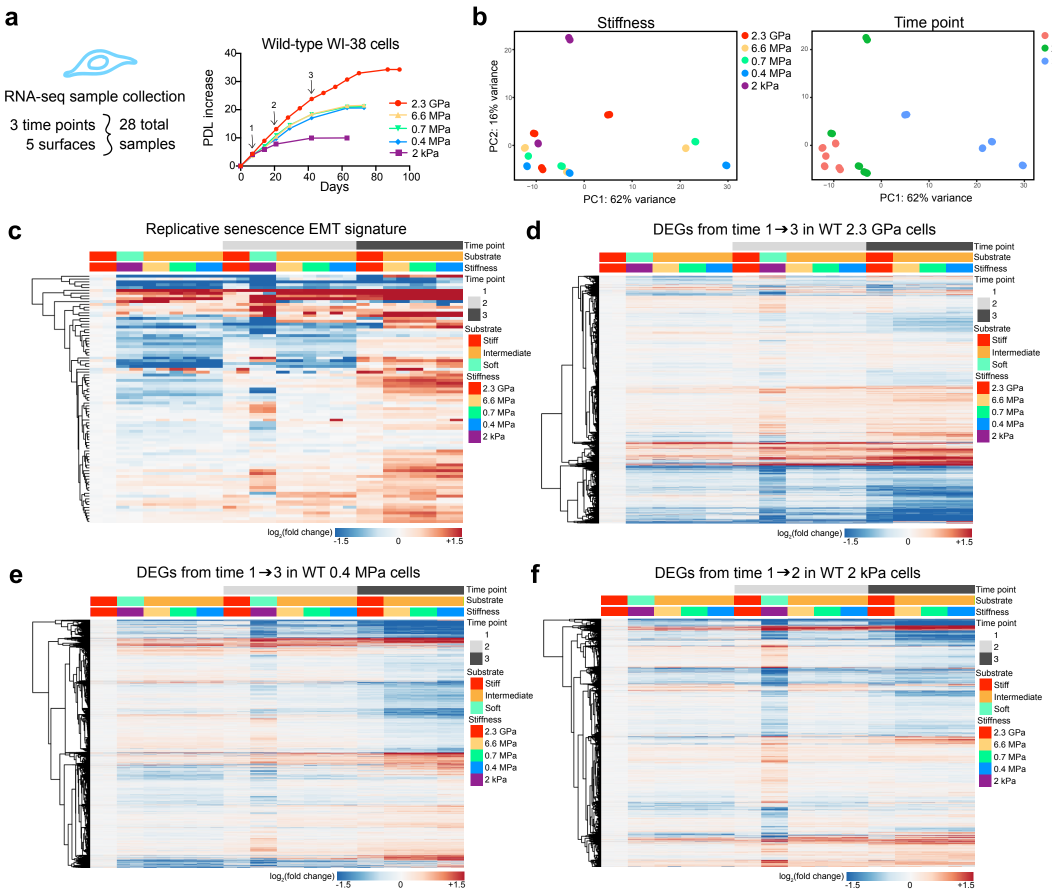

Figure S3: WI-38 cells cultured on a wide range of PDMS stiffnesses similarly regulate senescence-associated gene expression programs.

(a) Schematic of the (left) RNA-sequencing samples and (right) sample collection time points. (b) PC analysis showing PC1 and PC2 with samples colored by different variables. (c) Heatmap of the $\log_2$(fold change) of the EMT gene set (Supplemental Data File 2) previously identified[7] as upregulated in replicative senescence. (d-f) Heatmap of the $\log_2$(fold change) of all DEGs in wild-type cells grown on (d) 2.3 GPa, (e) 0.4 MPa, or (f) 2 kPa from time point 1 to time point 2 or 3 plotted across all samples across time.

a

Sample ID	Sample identity	Sample ID	Sample identity	Sample ID	Sample identity
1	WT1 2.3GPa time 1	13	WT1 2.3GPa time 4	25	WT1 2.3GPa time 7
2	WT2 2.3GPa time 1	14	WT2 2.3GPa time 4	26	WT2 2.3GPa time 7
3	hTERT1 2.3GPa time 1	15	hTERT1 2.3GPa time 4	27	hTERT1 2.3GPa time 7
4	hTERT2 2.3GPa time 1	16	hTERT2 2.3GPa time 4	28	hTERT2 2.3GPa time 7
5	WT1 0.5kPa time 1	17	WT1 0.5kPa time 4	29	WT1 0.5kPa time 7
6	WT2 0.5kPa time 1	18	WT2 0.5kPa time 4	30	WT2 0.5kPa time 7
7	hTERT1 0.5kPa time 1	19	hTERT1 0.5kPa time 4	31	hTERT1 0.5kPa time 7
8	hTERT2 0.5kPa time 1	20	hTERT2 0.5kPa time 4	32	hTERT2 0.5kPa time 7
9	WT1 32kPa time 1	21	WT1 32kPa time 4	33	WT1 32kPa time 7
10	WT2 32kPa time 1	22	WT2 32kPa time 4	34	WT2 32kPa time 7
11	hTERT1 32kPa time 1	23	hTERT1 32kPa time 4	35	hTERT1 32kPa time 7
12	hTERT2 32kPa time 1	24	hTERT2 32kPa time 4	36	hTERT2 32kPa time 7

b

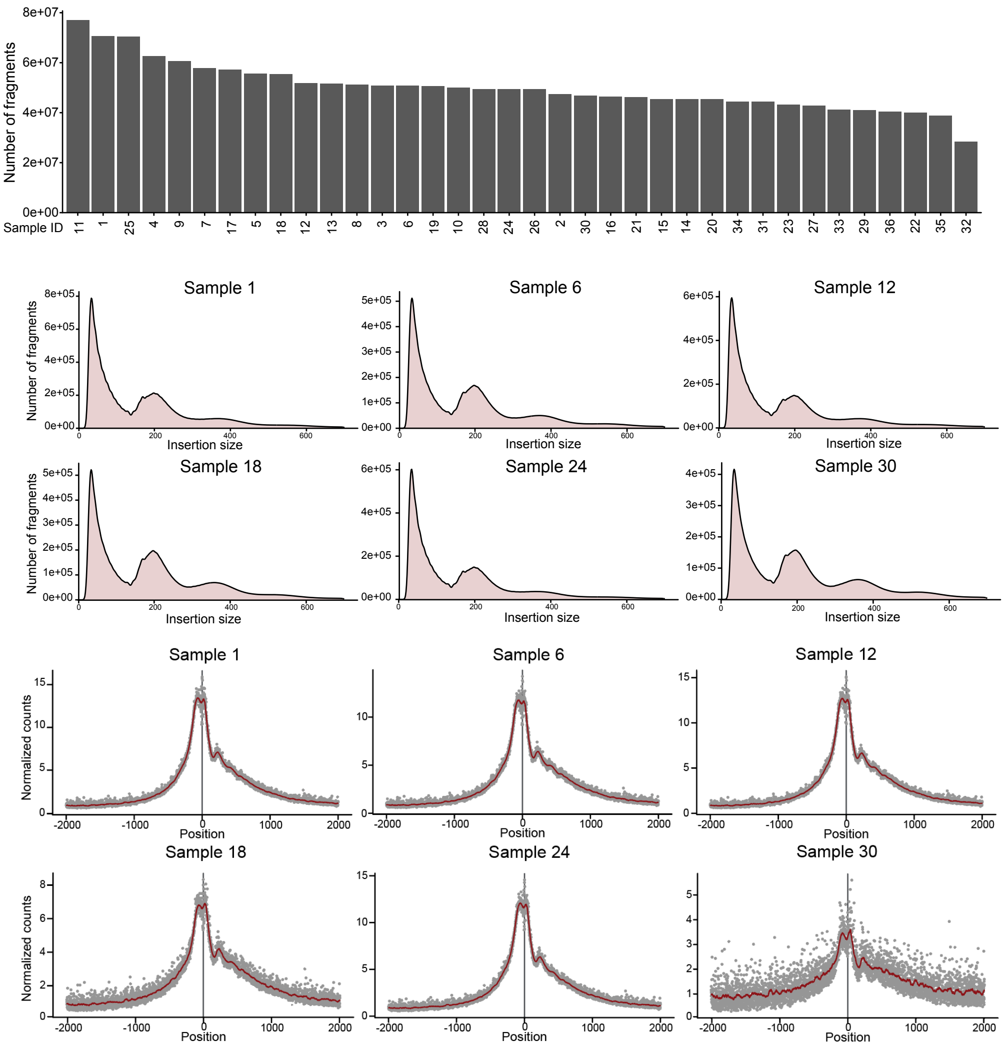

Figure S4: ATAC-sequencing library quality controls.

(a) Chart depicting the identity of ATAC-sequencing samples. (b) Bar graph displaying the fragment count of each ATAC-sequencing sample. (c) Fragment size distribution graphs of 6 different ATAC libraries shows a periodicity indicative of nucleosome bound DNA. (d) Plot of normalized counts per ATAC peak in the region ± 2 kilobases from the transcriptional start site to show enrichment of ATAC signal near the transcriptional start site of 6 different ATAC libraries.

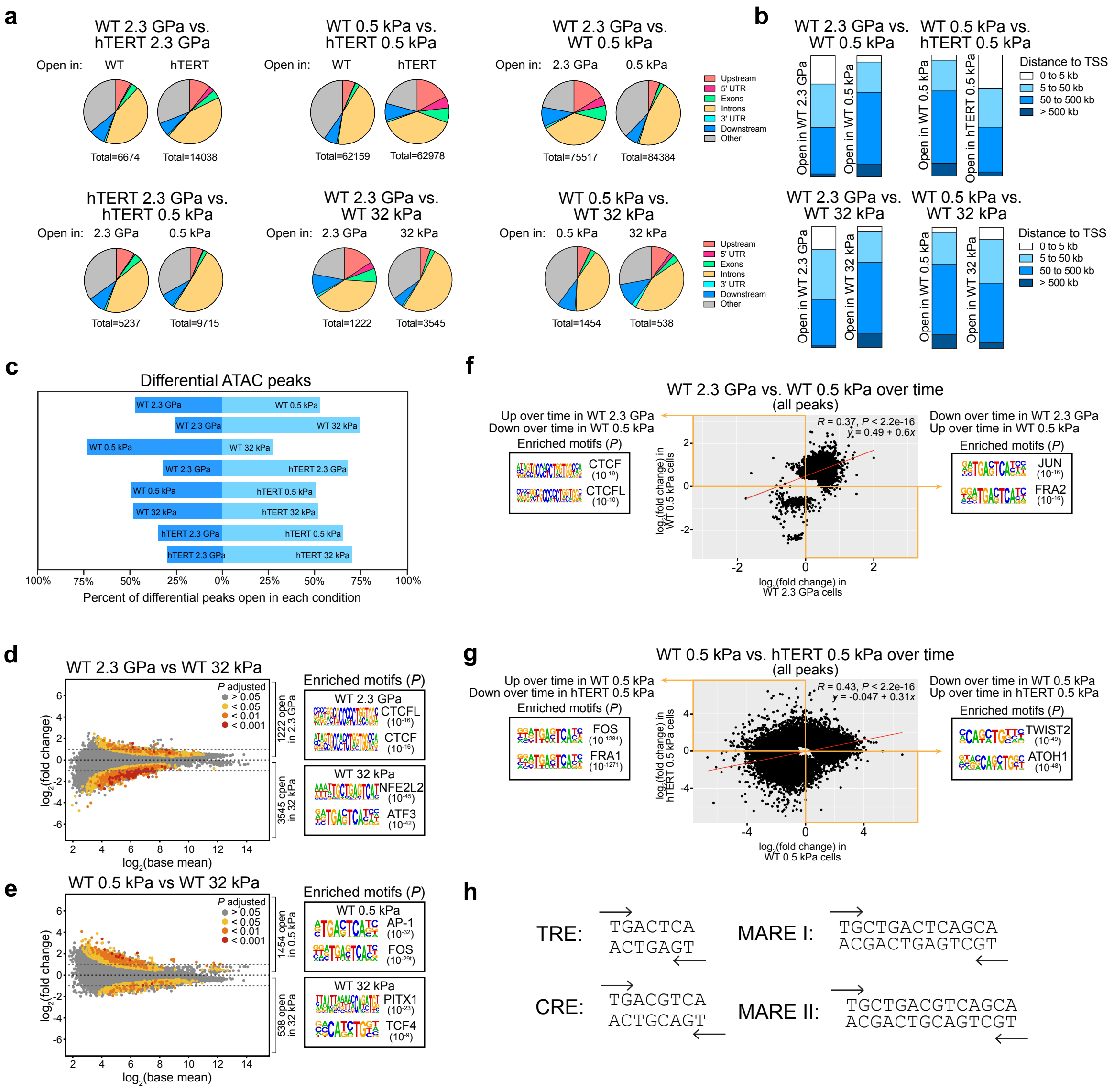

Figure S5: Patterns of chromatin accessibility change with matrix stiffness.
(a) Peak annotation of differentially accessible (DA) regions in the stated comparisons. UTR, untranslated region. (b) Distance of DA regions in the stated comparisons from the nearest transcriptional start site (TSS), plotted as a proportion of all DA regions. (c) Proportion of DA peaks (all peaks analysis) opening on each side of the stated comparisons. (d,e) Log_2 mean reads per region versus differential accessibility [log_2(fold change)] for peaks in the (d) wild-type 2.3 GPa versus wild-type 32 kPa comparison or (e) wild-type 0.5 kPa versus wild-type 32 kPa comparison. The top 2 transcription factor motifs enriched in DA regions identified by HOMER known motif analysis are shown. (f,g) All DA regions over time (time 1 versus time 7) in (f) wild-type cells on 2.3 GPa versus wild-type cells on 0.5 kPa or (g) wild-type cells on 0.5 kPa versus hTERT cells on 0.5 kPa were plotted as a function of the log_2(fold change) in each comparison. Yellow boxes highlight anti-correlated peaks. The top 2 transcription factor motifs enriched in the two anti-correlated peak sets identified by HOMER known motif analysis are

shown. (h) TPA response element (TRE), cAMP-response element (CRE), and MAF-recognition element (MAF) sequences[7,27].

P values for motif analysis were calculated using HOMER (d-g). Pearson correlation coefficients (*R*) and *P* values were calculated using ggpubr in R via stat_cor() and regression line equations were estimated using stat_regline_equation() (f,g).

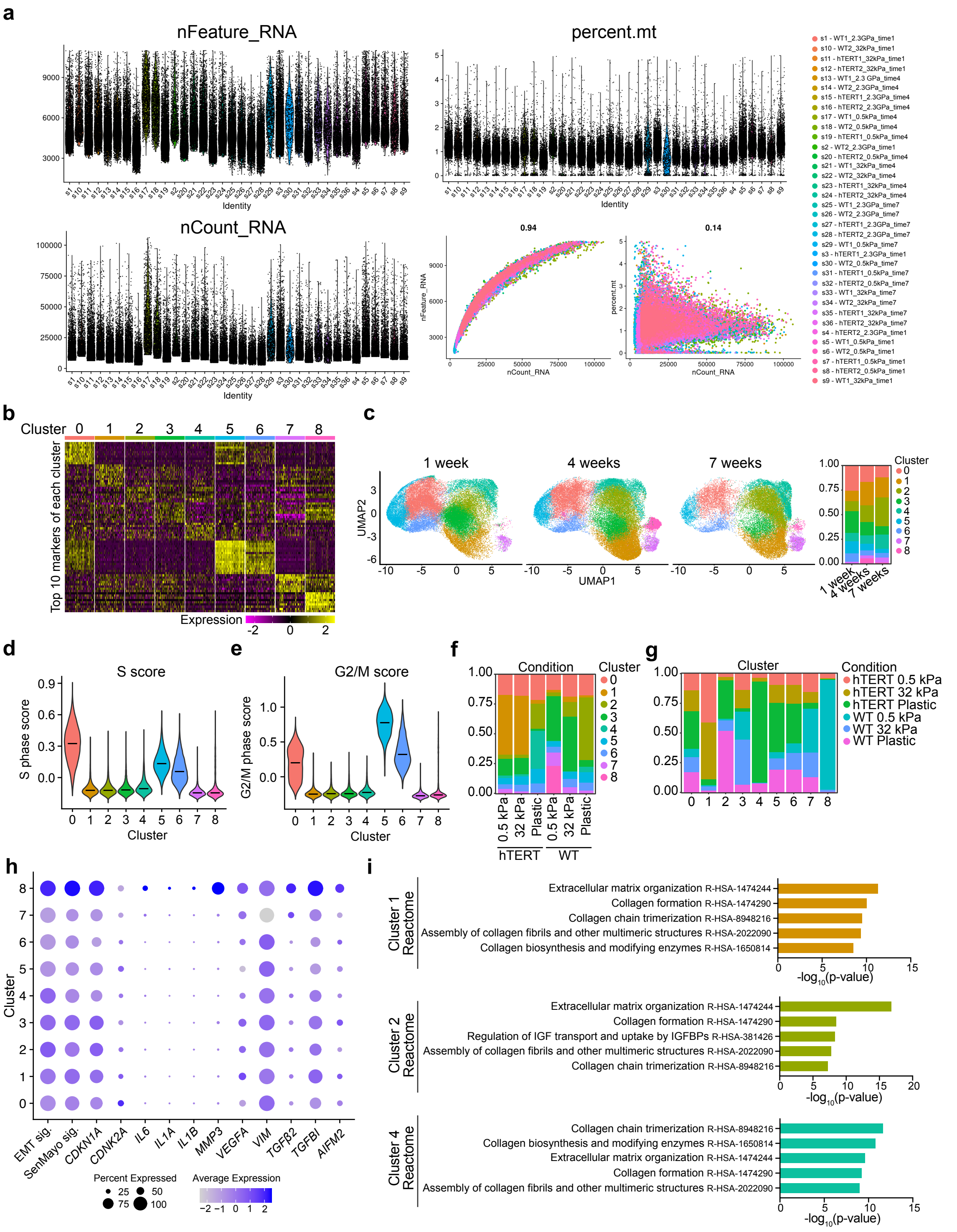

Figure S6: scRNA-sequencing reveals distinct cell clusters.
(a) Violin plots depicting the number of RNA features (nFeature_RNA), percent mitochondrial (mt)DNA (percent.mt), and the number of RNA counts (nCount_RNA) for individual cells in each scRNA-sequencing sample after filtering. Scatter plots show (left) nCount_RNA versus nFeature_RNA for individual cells and (right) nCount_RNA versus percent.mt for individual cells.

(b) Heatmap of the top 10 markers for each cluster calculated in R using Seurat via the FindAllMarkers() function. (c) (Left) UMAP plot from (Fig. 5b) split by time point. (Right) Bar graph displaying the relative proportion of cells in each cluster split by time point. (d,e) Violin plots showing the distribution of (d) S phase and (e) G2/M phase cell cycle scoring across clusters calculated in R using Seurat via the CellCycleScoring() function. (f) Bar graph displaying the relative proportion of cells in each cluster split by condition. (g) Bar graph displaying the relative proportion of cells in each condition split by cluster. (h) Dot plot showing the average expression of two senescence-associated gene expression programs (Supplemental Data File 2) across clusters and markers of senescence, the senescence-associated secretory program, and EMT. (i) Top 5 significantly enriched terms in cluster 1, 2, or 4 markers in the 2022 Reactome pathway database via Enrichr[33-35].
Pearson correlation coefficients (a) were calculated in R using Seurat via the FeatureScatter() function. Violin plots show the median.

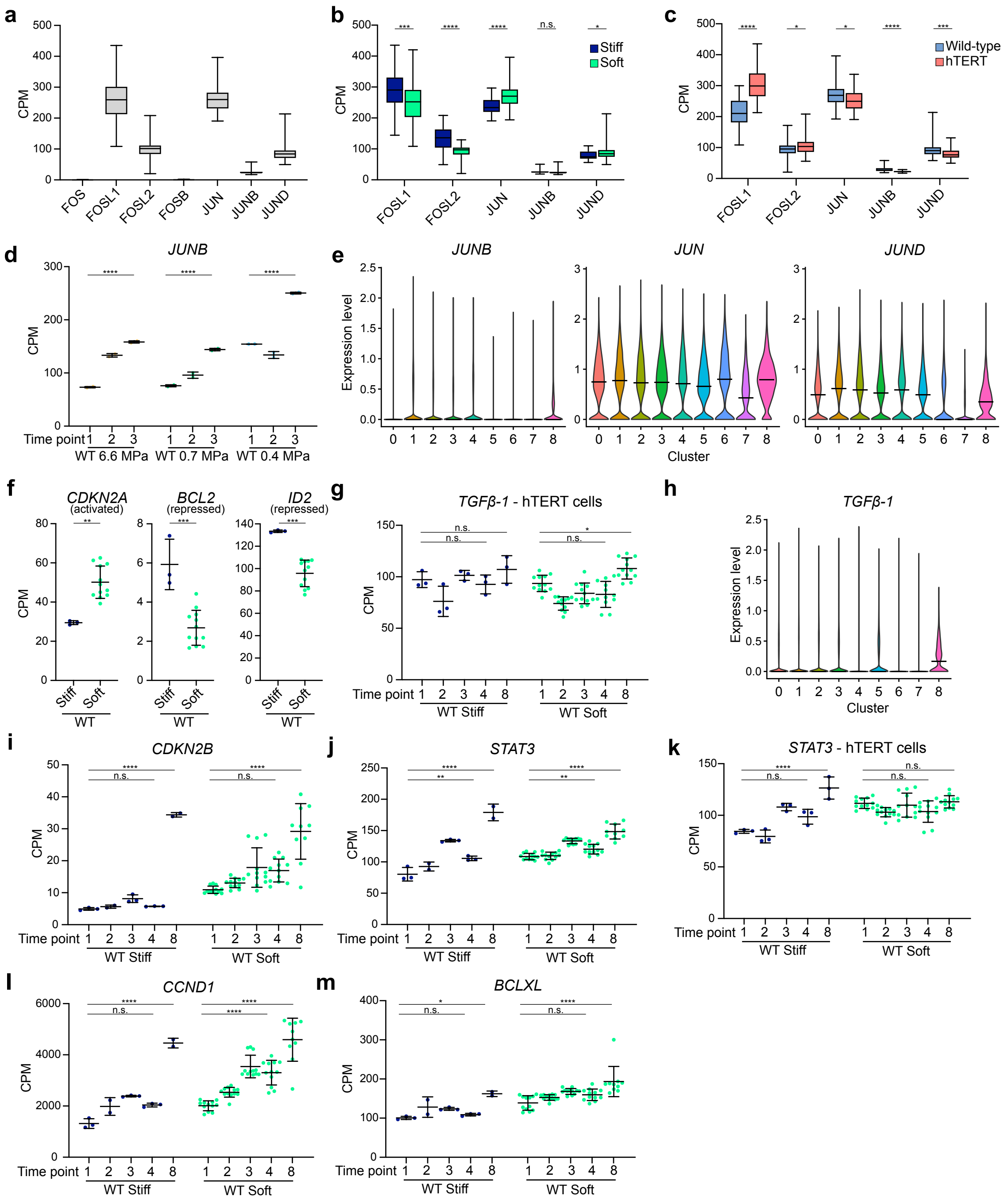
a
CPM
FOS
FOSL1
FOSL2
FOSB
JUN
JUNB
JUND
b
Stiff
Soft
n.s.
c
Wild-type
hTERT
d
JUNB
Time point
WT 6.6 MPa
WT 0.7 MPa
WT 0.4 MPa
e
JUNB
JUN
JUND
Expression level
Cluster
f
CDKN2A
(activated)
BCL2
(repressed)
ID2
(repressed)
Stiff
Soft
WT
g
TGFβ-1 - hTERT cells
WT Stiff
WT Soft
h
TGFβ-1
i
CDKN2B
j
STAT3
k
STAT3 - hTERT cells
l
CCND1
m
BCLXL

Figure S7: JUNB and JUNB regulators are induced with matrix softening.

(a-c) Box plots comparing the expression of JUN and FOS family members across all samples in the initial time course RNA-sequencing experiment. (d) *JUNB* expression across time in wild-type cells grown on substrates of intermediate stiffnesses. (e) Violin plots showing expression of JUN family members across clusters in the scRNA-seq experiment. (f) Expression of JUNB target genes in wild-type cells at time point 4. (g) Dot plots of *TGFβ-1* expression in hTERT cells. (h) Violin plot showing expression of *TGFβ-1* across clusters in the scRNA-seq experiment. (i,j) Dot plots of the indicated genes across time in wild-type cells. (k) Dot plot of *STAT3* expression in hTERT cells. (l,m) Dot plots of the indicated genes across time in wild-type cells.

Dot plots show mean ± standard deviation. Box plots show median ± 25th and 75th percentiles, with whiskers showing minimum to maximum. 178 replicates were used for (a). 45 and 133 replicates were used for stiff and soft substrates, respectively, in (b). 85 and 93 replicates were used for wild-type and hTERT cells, respectively, in (c). 2 replicates were used for each condition in (d). 3 and 12 replicates were used for stiff and soft substrates in (f). 3 replicates were used for the stiff time point 1, 3, and 4 conditions, 2 replicates were used for the stiff time point 2 and 8 conditions, 12 replicates were used for the soft time point 1, 2, 3, and 4 conditions, and 10 replicates were used for the soft time point 8 condition (g,i-m). *P* values were calculated by two-tailed Student's t-test (b,c,f) or ordinary one-way ANOVA with Tukey's multiple comparisons test (d, g, i-m). *$P < 0.05$, **$P < 0.01$, ***$P < 0.001$, ****$P < 0.0001$. n.s., not significant.

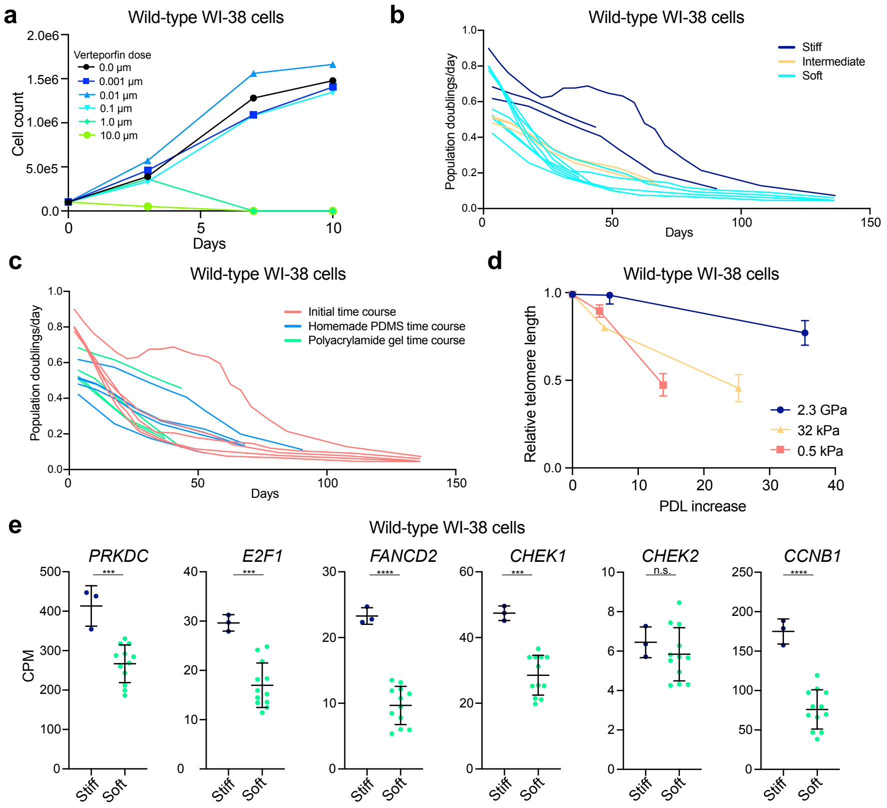
a
Wild-type WI-38 cells
Cell count
Days
Verteporfin dose
0.0 μm
0.001 μm
0.01 μm
0.1 μm
1.0 μm
10.0 μm
b
Wild-type WI-38 cells
Population doublings/day
Days
Stiff
Intermediate
Soft
c
Wild-type WI-38 cells
Population doublings/day
Days
Initial time course
Homemade PDMS time course
Polyacrylamide gel time course
d
Wild-type WI-38 cells
Relative telomere length
PDL increase
2.3 GPa
32 kPa
0.5 kPa
e
Wild-type WI-38 cells
PRKDC
E2F1
FANCD2
CHEK1
CHEK2
CCNB1
CPM
Stiff
Soft
n.s.

Figure S8: WI-38 cell culture dynamics and DNA damage response across conditions.
(a) Line graph displaying days in culture (x-axis) versus the total number of wild-type WI-38 cells (y-axis) when cells were treated with different concentrations of verteporfin. (b,c) Line graphs displaying days in culture (x-axis) versus the number of population doublings per day (y-axis) underwent by wild-type WI-38 cells over time across all experiments colored by (b) substrate stiffness or (c) experiment. (d) Line graph displaying the PDL increase (x-axis) versus the relative telomere length (y-axis) for wild-type WI-38 cells on the indicated surfaces. (e) Expression of DNA damage response markers at time point 4 in wild-type WI-38 cells on the indicated stiffnesses in the initial time course RNA-sequencing experiment.
2 replicates were performed for telomere measurements in (d). 3 and 12 replicates were used for stiff and soft substrates in (e). *P* values were calculated by two-tailed Student's t-test (e). ***$P < 0.001$, ****$P < 0.0001$. n.s., not significant.
